# Factors Associated with Statin Discontinuation Following Metabolic and Bariatric Surgery: A Retrospective Analysis of 2012–2021 Electronic Medical Records Network Data

**DOI:** 10.1007/s11695-024-07110-x

**Published:** 2024-02-22

**Authors:** Abdulrahman A Alsuhibani, Omar A Al-Obeed, Patricia R. Wigle, Mohammed M. Alsultan, Jeff J Guo, Alex C. Lin, Marepalli B Rao, Ana L. Hincapie

**Affiliations:** 1 Department of Pharmacy Practice, College of Pharmacy, Qassim University, Qassim, Buraidah, 51452 Saudi Arabia; 2 James L. Winkle College of Pharmacy, University of Cincinnati Academic Health Center, Cincinnati, OH 45267 USA; 3 Department of surgery, College of Medicine, King Saud University, Riyadh, Saudi Arabia; 4 Department of Pharmacy Practice, College of Clinical Pharmacy, Imam Abdulrahman Bin Faisal University, Dammam, 34212 Saudi Arabia; 5 Department of Environmental and Public Health Sciences, University of Cincinnati College of Medicine, Cincinnati, OH USA

**Keywords:** Obesity, Metabolic and bariatric surgery, Statin, RYGB, Sleeve gastrectomy, Weight loss, Cardiovascular disease

## Abstract

**Abstract:**

**Background:**

Bariatric surgery has been shown to improve hyperlipidemia, decreasing the need for statin medications. Although maintaining statin therapy post-surgery for those with a history of atherosclerotic cardiovascular disease (ASCVD) is advised, it is uncertain if discontinuation risks differ between those with and without ASCVD history.

**Aim:**

The study aims to analyze the rate and reasons for statin cessation post-bariatric surgery in the US using real-world data.

**Methods:**

Using the TriNetX electronic medical records network from 2012 to 2021, the study involved patients aged 18 or older on statins at the time of bariatric surgery. They were categorized into primary and secondary prevention groups based on prior ASCVD. Statin discontinuation was defined as a 90-day gap post the last statin dosage. The Cox model assessed factors influencing statin cessation.

**Results:**

Seven hundred and thirty-three statin users undergoing bariatric surgery were identified, with 564 (77%) in primary prevention. Six months post-surgery, 48% of primary prevention patients and 34.5% of secondary ones stopped statins. Primary prevention patients had a 30% higher likelihood of cessation compared to secondary prevention (hazard ratio, 1.30; 95% CI, 1.06–1.60) as shown by multivariable analysis.

**Conclusions:**

Post-bariatric surgery, primary prevention patients are more likely to discontinue statins than secondary prevention patients.

**Graphical Abstract:**

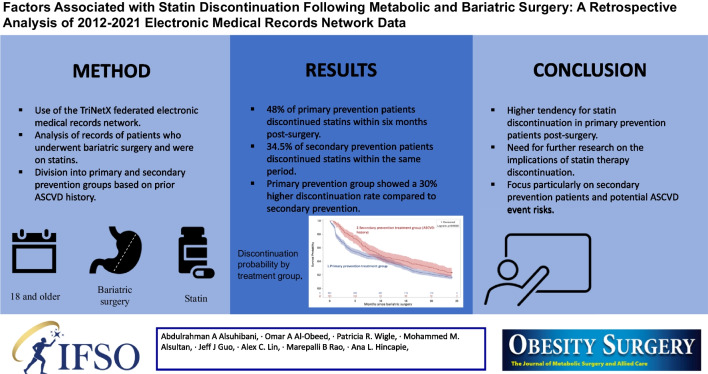

**Supplementary Information:**

The online version contains supplementary material available at 10.1007/s11695-024-07110-x.

## Introduction

Obesity is a major public health problem in the United States (U.S.), with an estimated 40% of the adult population being classified as individuals with obesity [1–3]. It is a significant risk factor for numerous chronic health conditions, including heart disease, type 2 diabetes, and certain types of cancer. The use of bariatric surgery as a treatment option for obesity has been increasing, reflecting its effectiveness in cases where obesity, a complex and multifactorial disease, has not been adequately managed through lifestyle modifications and medical treatments. The most performed bariatric surgical procedures include gastric bypass, sleeve gastrectomy (S.G.), and adjustable gastric banding. These procedures have been shown to result in substantial and sustained weight loss, as well as improvements in obesity-related comorbidities [4].

In recent years, the management of type 2 diabetes and obesity has evolved significantly, embracing a multimodal approach that combines surgical intervention with pharmacotherapy. This holistic approach is particularly relevant in light of the growing diabesity epidemic, which threatens global health and life expectancy. Surgical interventions, including various bariatric procedures, have been increasingly recognized not just for their direct impact on weight loss but also for their role in improving glucose homeostasis. These developments have been underscored by research such as the study outlined in a study published in 2015 [5], which highlights the effectiveness of combining surgical and medical therapies in treating patients with obesity and type 2 diabetes. By delving into the complex relationship between gut anatomy and glucose regulation, these approaches offer novel insights and potent therapeutic strategies for managing these intertwined conditions.

The efficacy and safety of bariatric surgery have been well documented in numerous clinical studies and systematic reviews [6–10]. Over the past two decades, the efficacy of bariatric surgery for weight reduction has been widely documented for patients with cardiovascular disease, as evidenced by several cohort and randomized controlled trials (RCTs) studies [11–15]. However, limited research has explored the impact of bariatric surgery on cardiovascular-related medication utilization [16–20]. The significant weight loss achieved through bariatric surgery has been shown to lead to the discontinuation of various medications, including those prescribed for hyperlipidemia [19–22]. The discontinuation of antihyperlipidemic drugs, specifically HMG-CoA reductase inhibitors, commonly referred to as statins, holds considerable importance. These medications serve a vital function in diminishing the likelihood of ASCVD in two distinct patient groups: primary prevention, which encompasses individuals without a prior history of ASCVD but possessing an elevated overall risk, and secondary prevention, which includes those with a documented history of ASCVD [23–26].

The discontinuation of anti-diabetic and anti-hypertensive medications after bariatric surgery to prevent the occurrence of hypoglycemia and hypotension respectively is a well-known practice [27–31]. However, the utilization of statins, a vital medication in reducing the risk of ASCVD, may require continued use post-surgery, especially in patients requiring subsequent ASCVD event prevention. According to established clinical recommendations, individuals who have experienced cardiovascular disease should persist in their utilization of statins for secondary preventive measures, irrespective of additional factors such as undergoing bariatric surgical procedures [26, 32, 33]. The European Association for the Study of Obesity’s Obesity Management Task Force for Post-Bariatric Surgery Medical Management Guidelines also recommends continuing lipid-lowering prescriptions utilization unless explicitly indicated [34].

Despite existing recommendations, a considerable gap persists in the literature regarding statin cessation among bariatric surgery patients. Our prior study [17] offered valuable insights into statin discontinuation rates in primary and secondary prevention populations using claims data. This study builds on our previous findings by leveraging a comprehensive, multi-center Electronic Health Records (EHRs) dataset with additional variables for enhanced robustness and generalizability. The primary objectives are to estimate the prevalence of statin therapy discontinuation post-bariatric surgery and to investigate the factors associated with this discontinuation. Importantly, this research aims to establish a baseline understanding of statin therapy management in the post-bariatric surgery population. The findings are intended to inform and guide future studies, which may be crucial for developing well-informed clinical recommendations and potentially influencing practice changes in this field.

## Methods

### Study Design

In this retrospective cohort study, we used the TriNetX (Cambridge, MA) database from January 2012 to December 2021. TriNetX is a Health Insurance Portability and Accountability Act of 1996 (HIPAA)-compliant health research data aggregator. It is a federated health research network platform with over 84 million patients’ EHRs from 69 healthcare organizations (HCOs) in the U.S., comprising primary care, hospitals, and specialty treatment providers. Most HCOs are large academic medical centers with inpatient and outpatient services. The TriNetX platform provides access to de-identified clinical data aggregated from participating or member HCOs. Both the patients and the HCOs who provide data remain anonymous [35–37].

Because only de-identified aggregated patient data were used, our study was exempt from requiring human subject approval from the Institutional Review Board (IRB) at the University of Cincinnati.

### Inclusion and Exclusion Criteria

In order to identify individuals who underwent bariatric surgery, we utilized current procedural terminology (CPT) codes (see Supplementary File 1). The index date was defined as the day of bariatric surgery. We identified the last statin prescription issued before the surgical procedure (index date) and added the corresponding days of supply, along with a 21-day grace period. If this calculated duration overlapped with the day of the surgical procedure, the patient was considered a statin user on the day of surgery. The statins considered in the analysis were approved by the U.S. Food and Drug Administration (FDA) in single and combination formulations. To be eligible for inclusion in the study, patients needed at least 1 year of data before their cohort entry.

Patients who had undergone a revision procedure within 12 months’ timeframe before their bariatric procedure (index date), as indicated by a specific CPT code, were excluded from the analysis (see Supplementary File 1).

### Study Groups

Patients who experienced ASCVD within 365 days prior to undergoing bariatric surgery (index date) were categorized as receiving “secondary prevention” treatment. The definition of ASCVD was derived from the codes listed in the International Classification of 10th Revision (ICD-10) (see supplementary file 2). This is in accordance with the 2018 ACC/AHA Multisociety Guidelines on the Management of Blood Cholesterol [38]. The scope of the definition includes several medical conditions such as stable or unstable angina pectoris, peripheral arterial disease (presumed to be caused by atherosclerosis), transient ischemic attack (TIA), stroke, any previous myocardial infarction (MI), and arterial revascularization, whether ischemic or non-ischemic in nature [39]. Patients who did not fulfill the criteria for secondary prevention were classified as receiving “primary prevention” treatment.

### Outcome

The outcome was statin discontinuation following the surgical procedure. This was characterized by the absence of a statin prescription refill after depleting the existing supply, in conjunction with a 90-day grace period. Upon meeting the defined outcome criteria, the date of discontinuation, signifying the outcome date, was established as the concluding date of the supply duration for the most recent prescription. Patients were followed up until the first occurrence of statin discontinuation, death, disenrollment, or the end of the study period in 2021, whichever occurred first. In this context, patients were censored at the earliest of these events.

### Covariates

**Fig. 1:**
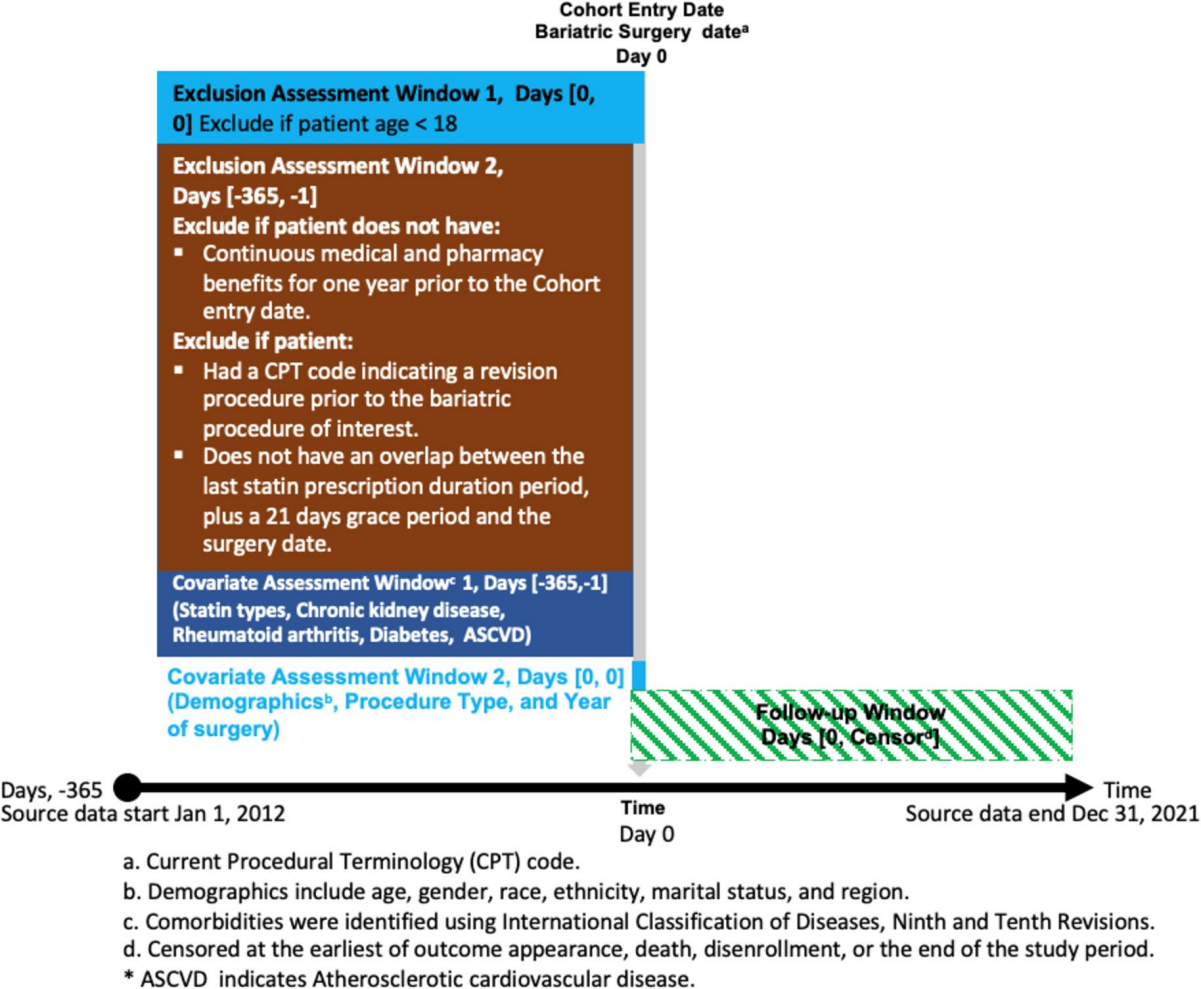
Study design for “Factors Associated with Statin Discontinuation Following Metabolic and Bariatric Surgery”

In our analysis, we considered several potential confounding factors, including age, sex, race, ethnicity, marital status, region, statin type at the time of surgery, type of bariatric surgery, alcohol use, and comorbidities, including diabetes mellitus, abnormal renal disease (including chronic kidney disease, acute kidney injury, glomerulonephritis, and nephrotic syndrome), cancer, and rheumatoid arthritis (RA). Also, the ASCVD condition that made a patient eligible for secondary prevention was also documented (Fig. 1).

### Statistical Analysis

Characteristics of the study population were represented as means (± standard deviation [SD]) for continuous variables and as percentages and frequencies for categorical variables. Disparities between the two study groups were examined using chi-square tests for categorical variables and *t*-tests for continuous variables. The aggregate count, percentage, and SD related to the cessation of statin usage after bariatric surgery were displayed and categorized by primary and secondary prevention. Moreover, the proportion of patients who discontinued statin treatment 6 months post-bariatric surgery was documented and partitioned by the type of prevention. The duration until discontinuation, according to prevention type, was visually represented through Kaplan-Meier curves and classified by the specific statin and bariatric surgery types.

An unadjusted model and adjusted multivariable Cox proportional hazards model were employed to assess the confounding variables associated with statin discontinuation, using hazard ratios (H.R.) and 95% confidence intervals (CI). The model was performed with time-to-discontinuation as the outcome variable after employing Schoenfeld residuals to verify the proportional hazards assumption.

### Subgroup Analyses

The study proceeded to compare the rate of statin discontinuation between the two studied cohorts, accounting for the type of bariatric surgery procedure (Roux-en-Y gastric bypass (RYGB), laparoscopic adjustable gastric banding (LAGB), S.G., biliopancreatic diversion (BPD-DS)), using H.R. and 95% CI. Furthermore, patients over the age of 40 and diagnosed with diabetes in the primary prevention treatment group were considered a distinct group, as the 2018 Blood Cholesterol Management Guideline recommended against discontinuing statin therapy for these patients, irrespective of other factors. The time-to-statin therapy discontinuation was illustrated using Kaplan-Meier curves, comparing this group with the other two groups [38]. The time-to-statin therapy discontinuation was illustrated using Kaplan-Meier curves, comparing this group with the other two groups.

Additionally, an examination of the interaction between the different surgical procedures and the classification of diagnosis groups was conducted to ascertain that the discontinuation of treatment did not result from channeling phenomena. This refers to the possibility that a greater percentage of individuals diagnosed with ASCVD may be assigned to RYGB in comparison to those without ASCVD, due to the more prominent decrease in weight associated with this procedure [40].

Version 9.4 of SAS software was used for all statistical analyses (SAS Institute Inc., Cary, NC, U.S.).

## Results

**Fig. 2:**
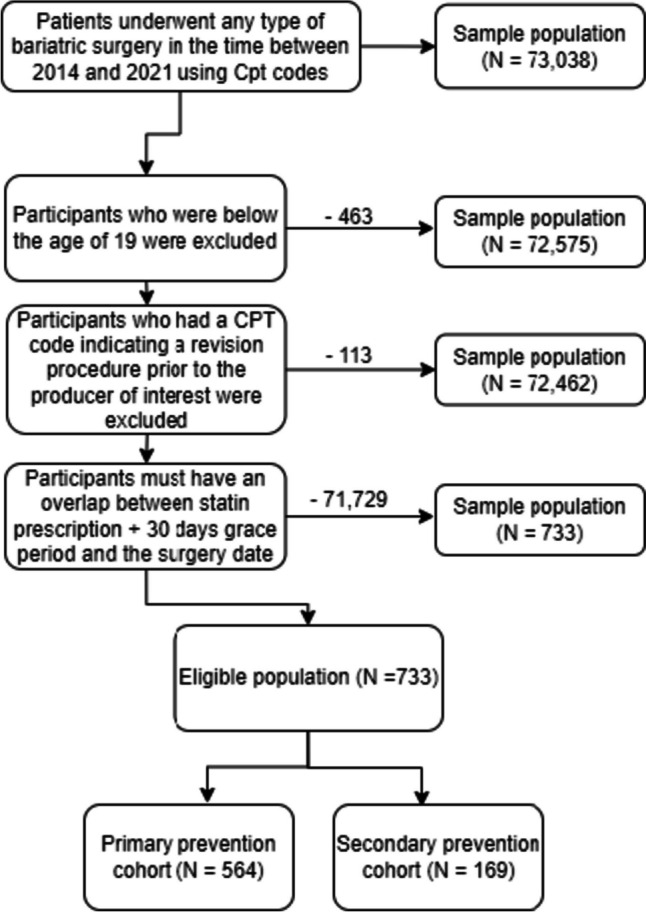
A flow chart of the sample selection

Our analysis comprised 733 adults who were on statin therapy during bariatric surgery. Of these, 564 (77%) were categorized under primary prevention, while 169 (23%) were part of the secondary prevention group. A diagram illustrating the sample selection can be found in Fig. 2 and Table 1.

**Table 1:** Baseline characteristics for primary versus secondary prevention treatment group

Characteristic (*N*= 733)	Primary prevention (no history of ASCVD)	Secondary prevention (history of ASCVD)	*P* value*
*N* (%)	564 (77)	169 (23)	
Age in years (mean, SD)	56 (10.5)	61 (9.1)	<.0001
Procedure type (*N*, %)			0.2490
(SG)	313 (55.5)	105 (62)	
(RYGB)	238 (42.2)	58 (34.4)	
(BPD-DS/WDS)	7 (1.2)	4 (2.4)	
(LAGB)	6 (1.1)	2 (1.2)	
Sex; female (*N*, %)	392 (71.8)	111 (66)	0.1524
Race (*N*, %)			0.0336
White	437 (77.5)	123 (72.8)	
Black or African American	87 (15.4)	38 (22.5)	
Unknown	32 (5.7)	7 (4.1)	
American Indian, Alaska or Asian	8 (1.4)	1 (0.6)	
Ethnicity (*N*, %)			0.4765
Not Hispanic or Latino	425 (75.3)	130 (76.9)	
Hispanic or Latino	99 (17.6)	27 (16.2)	
Unknown	40 (7.1)	12 (6.9)	
Marital status (*N*, %)			0.1712
Single	224 (39.7)	80 (47.3)	
Married	239 (42.4)	61 (36.1)	
Unknown	101 (17.9)	28 (16.6)	
Year of surgery (*N*, %)			0.0412
2014	51 (9)	13 (7.7)	
2015	59 (10.5)	18 (10.6)	
2016	90 (16)	20 (11.9)	
2017	92 (16.3)	19 (11.3)	
2018	89 (15.7)	31 (18.3)	
2019	79 (14)	29 (17.2)	
2020	49 (8.7)	23 (13.6)	
2021	55 (9.8)	16 (9.4)	
Region (*N*, %)			0.4108
Midwest	277 (49.2)	84 (49.7)	
South	183 (32.3)	58 (34.4)	
West	70 (12.4)	19 (11.2)	
Unknown	34 (6.1)	8 (4.7)	
Statin type at time of the surgery (*N*, %)			0.0329
Atorvastatin	289 (51.2)	102 (60)	
Simvastatin	141 (25)	30 (17.8)	
Pravastatin	79 (14)	19 (11.2)	
Rosuvastatin	34 (6)	11 (6.2)	
Fluvastatin/lovastatin/pitavastatin	21 (3.8)	7 (4.8)	
Alcohol use (*N*, %)	40 (7)	8 (4.9)	0.0834
Comorbidities (*N*, %)			
Diabetes mellitus	261 (46.3)	92 (54.4)	<.0001
Abnormal renal disease	54 (9.6)	45 (26.6)	<.0001
Cancer	24 (4.3)	16 (9.5)	0.0089
Rheumatoid arthritis	23 (4)	12 (6.8)	0.0331
ASCVD comorbidities (*N*, %)			
Angina	N/A	56 (33)	
Stroke	N/A	36 (21.5)	
Peripheral artery disease	N/A	37 (22.1)	
Myocardial infarction	N/A	20 (12)	
Arterial revascularization	N/A	17 (10.1)	
Transient ischemic attack	N/A	16 (9.7)	

*<0.05; atherosclerotic cardiovascular diseases (ASCVD), standard deviation (SD), Roux-en-Y gastric bypass (RYGB), laparoscopic adjustable banding (LAGB), sleeve gastrectomy (SG), biliopancreatic diversion with/without duodenal switch (BPD-DS/WDS)

The choice of statin used exhibited a significant disparity between the primary and secondary prevention groups (*p* = 0.0329). Specifically, 51.2% of the patients in the primary prevention group and 60% in the secondary prevention group utilized atorvastatin at the time of surgery, while 25% of the patients in the primary prevention group and 17.8% in the secondary prevention group utilized simvastatin at the time of surgery.

**Fig. 3:**
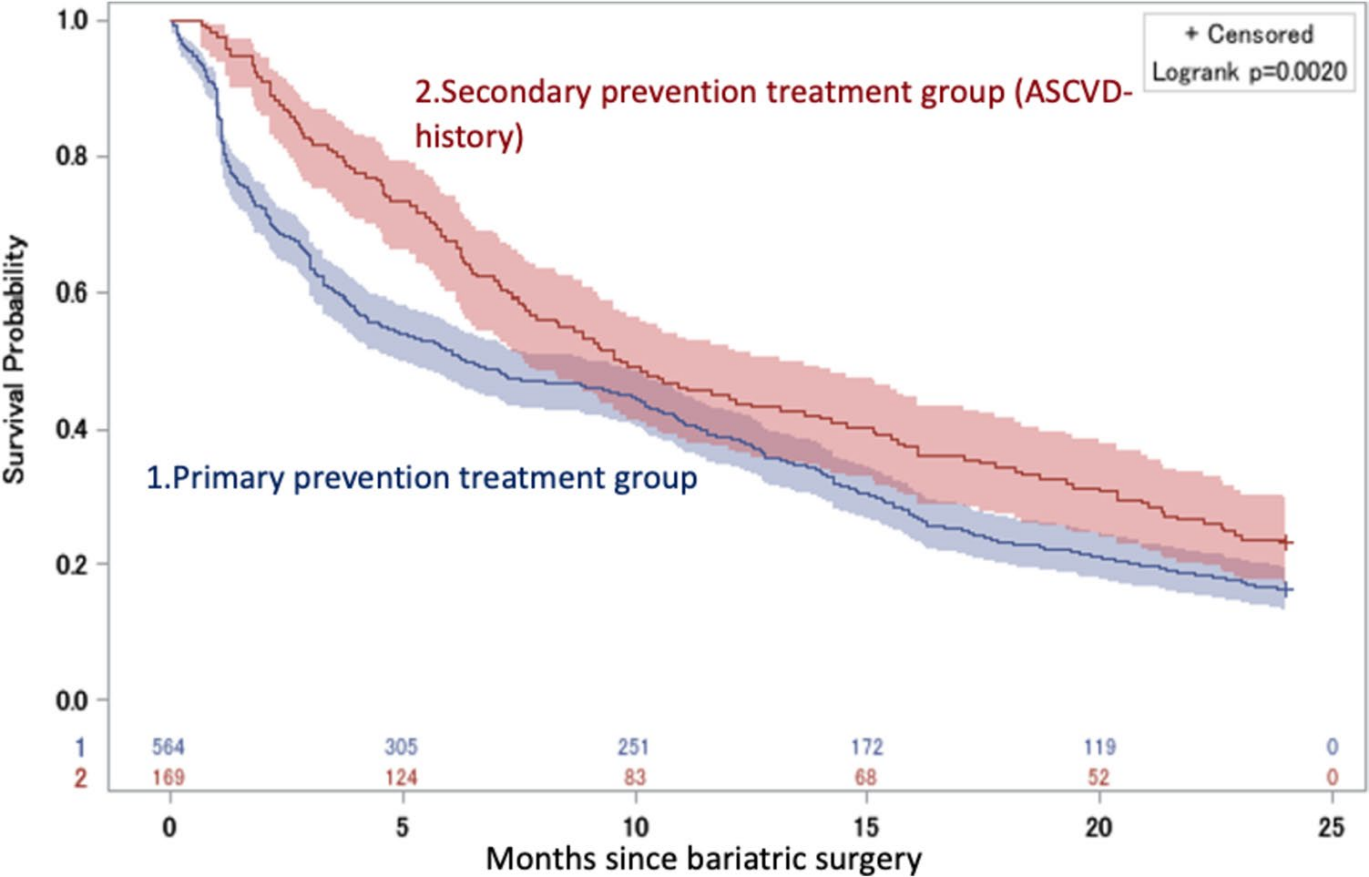
Discontinuation probability by treatment group. Discontinuation probability by treatment group, through 24 months. This figure shows the adjusted Kaplan-Meier curves for treatment discontinuation by treatment group from the time of bariatric surgery through 24 months of follow-up. The blue line represents patients from the primary prevention group, whereas the red line represents patients from the secondary prevention group

Kaplan-Meier analysis revealed a significant difference in the likelihood of statin discontinuation between the two cohorts (Fig. 3). Six months post-surgery, 48% of primary prevention patients and 34.5% of secondary prevention patients had discontinued statin usage. At the 1-year mark, these rates increased to 59.5% and 54% for primary and secondary prevention groups, respectively. The adjusted Kaplan-Meier curves, representing treatment discontinuation for all subjects from the point of bariatric surgery through 24 months of subsequent follow-up can be found in the online appendices in Supplementary Information Appendix A.

**Fig. 4:**
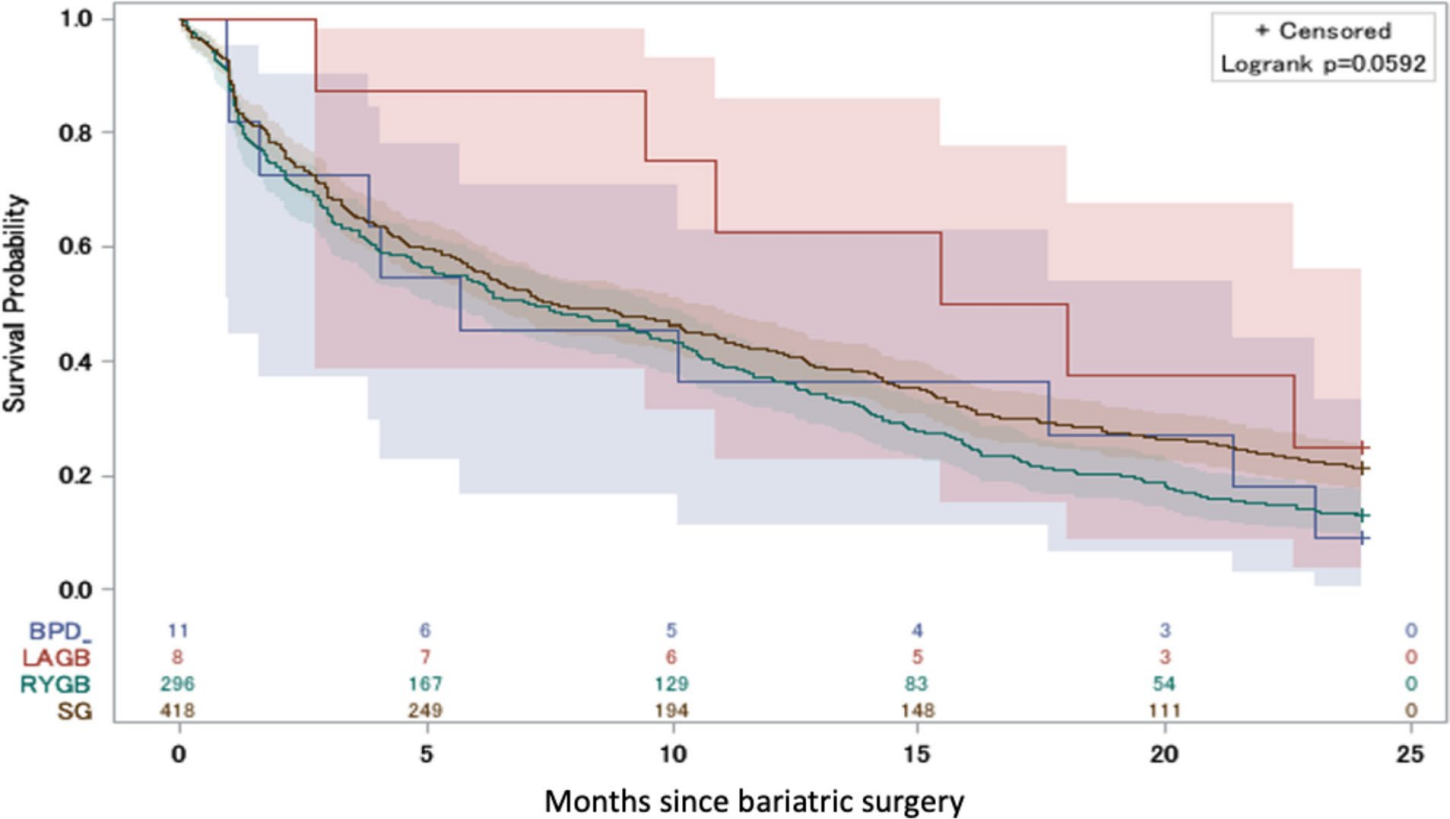
Discontinuation probability by the type of procedure. The figure shows the Kaplan-Meier estimates for medication discontinuation from the time of bariatric surgery through 24 months of follow-up stratified by procedure type

Upon categorizing patients by bariatric surgery types, the Kaplan-Meier estimates revealed substantial variations in statin therapy discontinuation rates across different procedures (Fig. 4). Individuals who underwent RYGB discontinued statin therapy sooner than those who had S.G. or LAGB. Six months post-surgery, the likelihood of ceasing statin therapy was 49% for RYGB patients and 45% for S.G. patients. Additionally, at 1 year, the disparity in statin discontinuation rates between these two groups began to expand (Fig. 4).

Table 2 outlines the factors influencing statin therapy discontinuation after bariatric surgery. After adjusting for covariates, primary prevention patients exhibited a 30% higher discontinuation rate than secondary prevention patients (HR 1.30, 95% CI 1.06–1.60). Additionally, the unadjusted Cox proportional hazard model revealed a 35% difference in discontinuation rates between the groups, as detailed in the online supplementary file. Although disparities in statin therapy discontinuation probabilities were observed across different demographic factors, none were statistically significant.

**Table 2:** Multivariable Cox proportional hazard model to identify predictors of medication discontinuation

Parameter	Hazard ratio	95% hazard ratio confidence limits		Pr >\Chi sq**
Treatment group (ASCVD)*	1.304	1.062	1.601	0.0112
Age group (>40)*	1.290	0.885	1.879	0.1850
Sex (F)*	1.042	0.868	1.249	0.6604
Statin types, versus (atorvastatin)*				
Fluvastatin/lovastatin/pitavastatin	1.169	0.745	1.837	0.4968
Pravastatin	1.304	1.016	1.674	0.0372
Rosuvastatin	1.005	0.708	1.427	0.9770
Simvastatin	1.237	1.006	1.522	0.0436
Procedure types, versus (SG)*				
BPD	1.529	0.800	2.925	0.1990
LAGB	0.708	0.309	1.623	0.4150
RYGB	1.244	1.046	1.480	0.0134
Race, versus (Black or African American)*				
American Indian, Alaska or Asian	0.870	0.361	2.094	0.7553
White	1.014	0.809	1.270	0.9033
Unknown	1.122	0.713	1.764	0.6184
Ethnicity, versus (not Hispanic or Latino)*				
Hispanic or Latino	1.076	0.833	1.389	0.5748
Unknown	1.411	0.873	2.280	0.1603
Marital status, versus (single)*				
Married	1.043	0.868	1.253	0.6550
Unknown	0.636	0.277	1.459	0.2855
Region, versus (South)*				
Midwest	1.164	0.946	1.432	0.1512
Unknown	1.927	0.734	5.056	0.1829
West	1.691	0.714	4.009	0.2326
Comorbidities				
Abnormal renal disease	1.052	0.811	1.366	0.7008
RA rheumatoid arthritis	0.838	0.539	1.304	0.4338
DM	0.685	0.580	0.810	<.0001
Alcohol use	1.190	0.724	1.954	0.4925
Cancer	0.849	0.582	1.239	0.3964

*Indicate the reference group; **<0.05

Treatment group (primary versus secondary prevention treatment group); *RYGB* Roux-en-Y gastric bypass, *LAGB* laparoscopic adjustable banding, *SG* sleeve gastrectomy, *BPD* biliopancreatic diversion/with and without duodenal switch, *RA* rheumatoid arthritis, *DM* diabetes mellitus

Categorical variables have an interpretation for each level as compared with a reference group. For example, an individual who underwent RYGB procedure has about 26% more hazard for statin therapy discontinuation compared with an individual who underwent SG procedure

Patients who underwent RYGB procedure (H.R. 1.24, 95% CI 1.04–1.48) displayed a considerably higher likelihood of therapy discontinuation compared to those who underwent sleeve gastrectomy procedure. Significant discontinuation differences were not found among various statin types, except for simvastatin in comparison to atorvastatin (H.R. 1.23, 95% CI 1.01–1.52) and pravastatin in comparison to atorvastatin (H.R. 1.30, 95% CI 1.02–1.67) (online supplementary file). Furthermore, patients with diabetes mellitus were significantly less likely to discontinue statin therapy (H.R. 0.68, 95% CI 0.58–0.81), while no significant findings were observed for abnormal renal disease, rheumatoid arthritis, alcohol use, or cancer.

The outcomes of the subgroup evaluations indicate that the rate of statin treatment discontinuation in the primary prevention cohort, compared to the secondary prevention cohort among individuals who received the S.G. procedure, was 42% (H.R. 1.42, 95% CI 1.09–1.86). In contrast, the results of data analysis for patients who underwent RYGB, LAGB, or BPD interventions did not yield statistically significant findings (online supplementary file).

**Fig. 5:**
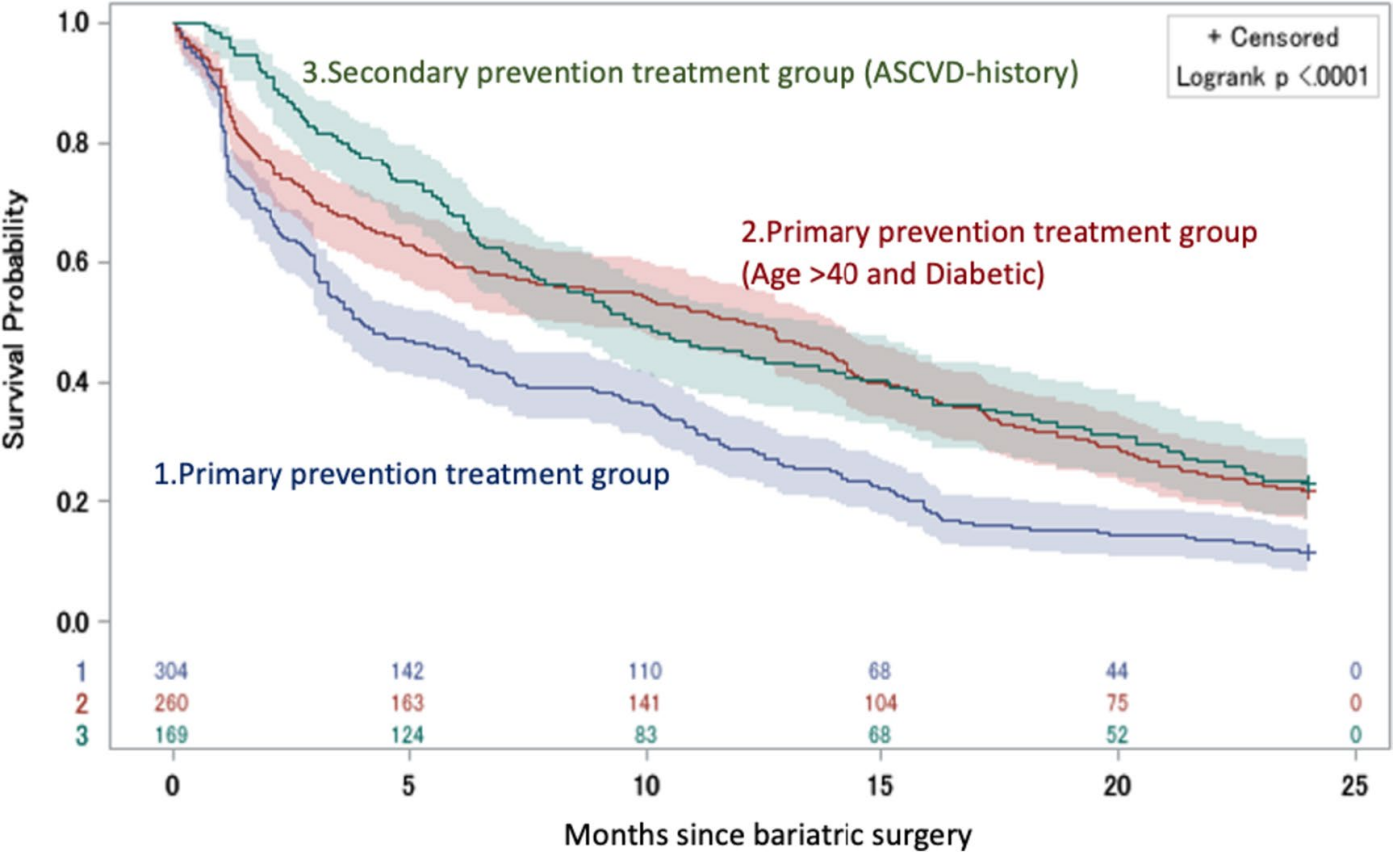
Discontinuation probability by treatment group. Discontinuation probability by treatment group, through 24 months. This figure shows the adjusted Kaplan-Meier curves for treatment discontinuation by treatment group from the time of bariatric surgery through 24 months of follow-up. The blue line represents patients from the primary prevention group, red line represents patients from the primary prevention group aged >40 and diagnosed with diabetes mellitus, whereas the green line represents patients from the secondary prevention group

Kaplan-Meier curves were employed to compare time-to-statin therapy discontinuation for patients over 40 years old diagnosed with diabetes mellitus to other groups. The probability of time-to-statin therapy discontinuation was higher for these patients than for patients using statins as secondary prevention within the first seven months post-surgery. However, after 7 months, the curves crossed over and aligned with the secondary prevention group (Fig. 5). The outcomes of the interaction analysis imply that no meaningful correlation exists between the factors in question. To elaborate, the procedure type and diagnostic group independently impact the rates of statin treatment discontinuation; yet, their combined effect lacks statistical significance.

## Discussion

The current investigation indicates a significant 30% difference in the adjusted hazard ratio for discontinuing statin therapy between the studied cohorts. Employing the Kaplan-Meier approach, our analysis revealed that 52% and 65.5% of patients maintained statin therapy for primary and secondary prevention, respectively, 6 months post-bariatric surgery, with corresponding figures of 40.5% and 46% at 12 months.

Our results suggest that there may be differences in discontinuation rates across various bariatric surgery techniques, although not all of these findings were statistically significant. For instance, patients receiving LAGB appeared to have a 70% lower likelihood of ceasing statin therapy compared to those undergoing RYGB, but the observed difference was not statistically significant (*p* = 0.4150). One possible explanation for this trend, albeit not confirmed by our data, could be the less substantial weight loss achieved by LAGB compared to other procedures, which might reduce the perceived necessity of discontinuing statin therapy [40, 41]. Furthermore, a higher discontinuation rate for statin therapy was observed in patients undergoing RYGB compared to S.G. by 24% (*p* = 0.0134). This could be due to the superior weight loss and comorbidity resolution associated with RYGB, implying that discontinuation is likely motivated by clinical improvements such as weight loss, type 2 diabetes mellitus (T2DM) resolution, and enhanced lipid profiles, as RYGB outperforms S.G. in these domains [42, 43]. Maciejewski et al. [40] reported that RYGB patients experienced 9.7% greater weight loss (95% CI, 0.8–18.6%) than S.G. patients 4 years post-surgery. However, it is crucial to consider that these differences might also be influenced by clinical bias, where practitioners might expect different outcomes from each surgery type, affecting their decisions on statin therapy continuation. For example, the lower discontinuation rates after LAGB and SG could reflect an expectation of inferior outcomes compared to RYGB and BPD, thereby influencing clinical decisions. Further research is needed to explore these dynamics comprehensively.

Previous research examining lipid-lowering medication use following bariatric surgeries has identified substantial rates at which individuals stopped taking statins, which were consistent with our findings [17, 19, 21, 22]. Schauer et al. [44] observed declines in lipid-lowering drug usage of 39% and 59% for S.G. and RYGB patients, respectively, after 12 months. Similarly, Segal et al. found that medication use for dyslipidemia decreased by 54% and 59% for T2DM and non-T2DM patients, respectively, 12 months post-surgery [21]. Furthermore, Maciejewski et al.’s study [19] of 298 veterans with hyperlipidemia who performed bariatric surgery discovered that approximately 40% discontinued lipid-lowering medications within a year.

The observed reduction in statin use among primary prevention patients appears justifiable, considering the probable decrease in ASCVD risk factors accompanying postoperative weight loss. However, the decline in statin use in secondary prevention patients following bariatric surgery raises potential concerns. Per clinical guidelines [26, 32], it is not advised for patients with a history of ASCVD to cease statin treatment, irrespective of their weight, given the potential for reoccurrence of ASCVD-related events. In our study, 54% of secondary prevention patients ceased statin therapy within 1-year post-surgery. The consequences of statin discontinuation after the surgery in individuals with a history of ASCVD and the subsequent risk of MI or stroke remain uncertain.

Moreover, we examined factors potentially associated with statin therapy discontinuation following bariatric surgery. Patients with diabetes mellitus exhibited lower rates of statin therapy discontinuation compared to other patients. We postulate that these patients receive more extensive care than their counterparts without diabetes, likely owing to a heightened concurrent ASCVD risk [42, 45].

### Strengths and Limitations

Several limitations and strengths of this study should be acknowledged. Firstly, the study is subject to the inherent limitations of retrospective analyses relying on routinely collected data. The quality and comprehensiveness of the data are contingent on how it was recorded within the database. In this context, TriNetX data are restricted to EHRs obtained during standard clinical practice without supplementary chart review data. As TriNetX data were not explicitly gathered for research purposes, potential miscoding of diagnoses and clinical events might occur.

Secondly, although EHR databases may provide more up-to-date, comprehensive, and accurate patient health information than claims data, they only encompass data from participating healthcare systems within the research network and exclude information from other doctors or providers. Thirdly, while certain demographic variables such as insurance status and income were not available in the dataset, body mass index (BMI) data was included but exhibited significant gaps. Specifically, the absence or incompleteness of BMI data at the outcome time was markedly higher than at the index date, which could compromise the accuracy of any average value derived from this dataset. Consequently, we decided not to include BMI in our analysis due to the high level of missing data and its potential impact on the reliability of our findings. We acknowledge this limitation, and future research should aim to include complete BMI data to better understand its influence on the outcomes. Furthermore, the available data preclude the ability to account for mortality as a potential censoring event, a factor particularly pertinent for individuals in the secondary prevention treatment group. Nonetheless, the proportion of censored patients was found to be analogous between the primary and secondary prevention treatment cohorts. Lastly, we could not distinguish between provider-initiated discontinuation, medication non-adherence, or patient self-discontinuation. Our definition of statin discontinuation as a 90-day gap following the last day of supply for the most recent statin prescription minimizes the likelihood of non-adherence impacting our results. We also anticipate that instances of self-discontinuation, attributable to factors such as expenses or adverse events like myalgia, would exhibit a comparable frequency between the two cohorts under study [46].

Strengths of this study encompass the utilization of recent, multi-year EHR data obtained from the TriNetX network, which is not limited to patients receiving health coverage from private or public insurers. The application of robust epidemiological methods helped address methodological challenges. Through the evaluation of varying risks associated with statin treatment cessation between primary and secondary prevention cohorts, as well as the extension of the follow-up duration, this investigation contributes essential understanding to the field of bariatric surgery, specifically concerning the discontinuation of statin therapy, particularly for individuals with a prior history of ASCVD.

## Conclusions

Our research findings suggests an elevated prevalence of statin treatment discontinuation after bariatric surgery in the primary prevention cohort as opposed to the secondary prevention group. Furthermore, we recognized variables correlated with the discontinuation of statin therapy. Further research is warranted to investigate the potential implications of inappropriate statin therapy discontinuation, particularly among patients undergoing secondary prevention treatment, and the associated risk of subsequent ASCVD events.

### Supplementary Information

Supplementary file 1 (DOCX 301 kb)

Supplementary file 2 (DOCX 32 kb)

## Abbreviations

BPD: Biliopancreatic diversion
LAGB: Laparoscopic adjustable gastric banding
RYGB: Roux-en-Y gastric bypass
S.G.: Sleeve gastrectomy
