# Supplementary material for: Factors Associated with Statin Discontinuation Following Metabolic and Bariatric Surgery: A Retrospective Analysis of 2012–2021 Electronic Medical Records Network Data
Source: Obes Surg. 2024 Feb 22;34(4):1267–78. doi: 10.1007/s11695-024-07110-x (PMC11026246; doi:10.1007/s11695-024-07110-x)
Supplement: Supplementary file 1 — (DOCX 301 kb) [file 11695_2024_7110_MOESM1_ESM.docx]

**Factors Associated with Statin Discontinuation Following Metabolic and Bariatric Surgery: A Retrospective Analysis of 2012-2021 Electronic Medical Records Network Data**

**Appendices**

Appendix-A


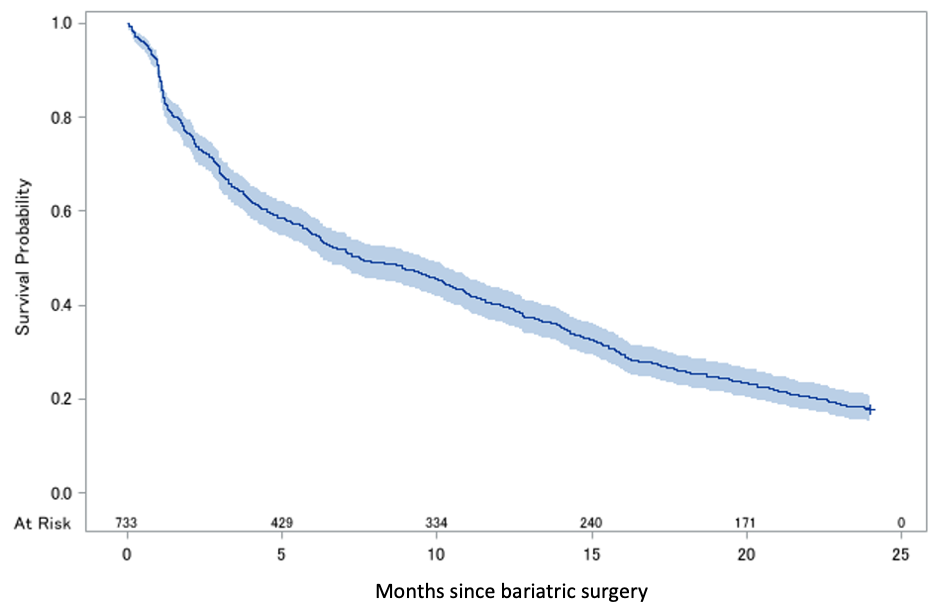


Discontinuation probability across all participants. Discontinuation probability of overall, through 24 months. This figure shows the adjusted Kaplan-Meier curves for treatment discontinuation across all participants from the time of bariatric surgery through 24 months of follow-up.

Appendix-B


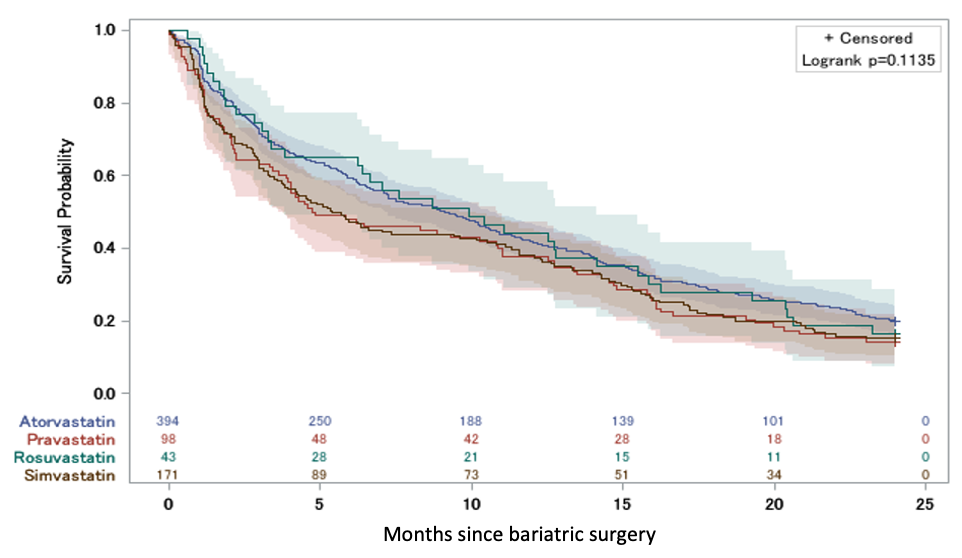


Discontinuation probability by statin type. The figure shows the Kaplan-Meier estimates for medication discontinuation from the time of bariatric surgery through 24 months of follow-up stratified by procedure type.

Appendix-C

The results of the subgroup analyses stratified by the type of bariatric surgery

| Analysis of Maximum Likelihood Estimates | | | | | |
| --- | --- | --- | --- | --- | --- |
| Parameter | Pr > ChiSq | Hazard Ratio | 95% Hazard Ratio Confidence Limits | | Bariatric surgery type |
| Treatment group ( ASCVD)* | 0.7047 | 1.066 | 0.765 | 1.487 | RYGB |
| Treatment group ( ASCVD)* | 0.3942 | 4.439 | 0.144 | 136.914 | LAGB |
| Treatment group ( ASCVD)* | 0.0090 | 1.429 | 1.093 | 1.868 | SG |

*Indicate the reference group; RYGB, Roux-en-Y gastric bypass; LAGB, Laparoscopic adjustable banding; SG, Sleeve gastrectomy.

Appendix-D

The Unadjusted Cox Proportional Hazard Model

| Parameter | Hazard Ratio | 95% Hazard Ratio Confidence Limits | | Pr > ChiSq** |
| --- | --- | --- | --- | --- |
| Treatment group (ASCVD)* | 1.356 | 1.117 | 1.647 | 0.0021 |

*Indicate the reference group; ** <0.05

Appendix-E

Diagnosis and Procedure Codes for Bariatric Surgery and Obesity

| Variable | Codes |
| --- | --- |
| Open Roux-en-Y gastric bypass (RYGB) | CPT-4: 43846, 43847  ICD-9-CM: 44.31, 44.39  ICD-10: 0D16078, |
| Laparoscopic Roux-en-Y gastric bypass (RYGB) | CPT-4: 43644, 43645  ICD-9-CM: 44.38  ICD-10: 0D16479, 0D1647A, 0D164J9, 0D164JA, 0D164K9, 0D164KA, 0D164Z9,  0D164ZA, 0D164ZB |
| Open Sleeve gastrectomy (SG) | CPT: 43843,  ICD-9-CM: 43.89, 44.69  ICD-10: 0DQ60ZZ |
| Laparoscopic Sleeve gastrectomy (SG) | CPT: 43775  ICD-9-CM: 43.82  ICD-10: 0DB64Z3 |
| Laparoscopic adjustable gastric band (AGB) | CPT-4: 43770, S2082  ICD-9: 44.95  ICD-10: 0DV64CZ |
| Laparoscopic single anastomosis duodenal-ileal bypass with sleeve (SADI-S) | CPT-4: 43999 |
| Biliopancreatic Diversion with Duodenal Switch (BPD/DS) or Gastric Reduction Duodenal Switch (BPD/GRDS) | CPT-4: 43845, (without Duodenal Switch)  ICD-9-CM: 45.91, 45.51, 43.89  ICD-10: 0D190Z9, 0DB60ZZ, 0DB80ZZ |
| Open Vertical-banded gastroplasty (VBG) | CPT: 43842 “Vertical-banded gastroplasty, not performed anymore”, VGB procedures are essentially no longer performed.  ICD-9-CM: 44.68  ICD-10: 0DQ64ZZ |
| Obesity | ICD-9-CM: 278.0x, 278.01, V77.8, V85.41, V85.42, V85.43, V85.44, V85.45,  ICD-10: E66.xx, Z68.4x |
| Morbid obesity | ICD-9: 278.01  ICD-10: E66.01 |

CPT-4, Current Procedure Terminology (American Medical Association, Chicago, IL),

ICD-9-CM, International Classification of Diseases, Ninth Revision, Clinical Modification,

ICD-10-CM, International Classification of Diseases, Tenth Revision, Clinical Modification.

Appendix-F

Procedure Codes Used to Identify Open and Laparoscopic Revisional Bariatric Surgery

| Description | Code | Code Type |
| --- | --- | --- |
| Laparoscopy, surgical, gastric restrictive procedure; removal of adjustable gastric restrictive device component only | 43772 | CPT-4 |
| Revision of gastrojejunal anastomosis (gastrojejunostomy) with reconstruction, with or without partial gastrectomy or intestine resection; with vagotomy | 43865 | CPT-4 |
| Revision of gastroduodenal anastomosis (gastroduodenostomy) with reconstruction; without vagotomy | 43850 | CPT-4 |
| Revision of gastroduodenal anastomosis (gastroduodenostomy) with reconstruction; with vagotomy | 43855 | CPT-4 |
| Laparoscopy, surgical, gastric restrictive procedure; revision of adjustable gastric restrictive device component only | 43771 | CPT-4 |
| Laparoscopy, surgical, gastric restrictive procedure; removal and replacement of adjustable gastric restrictive device component only | 43773 | CPT-4 |
| Revision of gastrojejunal anastomosis (gastrojejunostomy) with reconstruction, with or without partial gastrectomy or intestine resection; without vagotomy | 43860 | CPT-4 |
| Revision, open, of gastric restrictive procedure for morbid obesity, other than adjustable gastric restrictive device (separate procedure) | 43848 | CPT-4 |
| Laparoscopy, surgical, gastric restrictive procedure; removal of adjustable gastric restrictive device and subcutaneous port components | 43774 | CPT-4 |
| Gastric restrictive procedure, open; removal of subcutaneous port component only | 43887 | CPT-4 |
| Gastric restrictive procedure, open; revision of subcutaneous port component only | 43886 | CPT-4 |
| Gastric restrictive procedure, open; removal and replacement of subcutaneous port component only | 43888 | CPT-4 |
| Laparoscopic removal of gastric restrictive device(s) | 44.97 | ICD-9 |
| Laparoscopic revision of gastric restrictive procedure | 44.96 | ICD-9 |
| Open revision of RYGB | 44.5 | ICD-9 |

Appendix-G

List of all ICD-9/ICD-10 codes used to define/identity established ASCVD.

| ADA Class | ICD-10 |  |
| --- | --- | --- |
|  | I60 - I69 | Cerebrovascular Diseases |
| Stroke | I60 | Nontraumatic subarachnoid hemorrhage |
| Stroke | I61 | Nontraumatic intracerebral hemorrhage |
| Stroke | I62 | Other and unspecified nontraumatic intracranial hemorrhage |
| Stroke | I63 | Cerebral infarction |
| Stroke | I65 | Occlusion and stenosis of precerebral arteries, not resulting in cerebral infarction |
| TIA |  |  |
| Stroke | I66 | Occlusion and stenosis of cerebral arteries, not resulting in cerebral infarction |
| Stroke | I67.2 | Cerebral atherosclerosis |
| Stroke | I67.81  I67.82  I67.83  I67.84 | Acute cerebrovascular insufficiency  Cerebral ischemia  Posterior reversible encephalopathy syndrome  Cerebral vasospasm and vasoconstriction |
| Stroke | I69 | Sequelae of cerebrovascular disease |
| Stroke | R29.7 | National Institutes of Health Stroke Scale (NIHSS) score |
|  | I20 - I25 | Ischemic Heart Diseases |
| MI | I21 | ST elevation (STEMI) and non-ST elevation (NSTEMI) myocardial infarction |
| MI | I22 | Subsequent ST elevation (STEMI) and non-ST elevation (NSTEMI) myocardial infarction |
| ACS | I24 | Other acute ischemic heart diseases |
| MI | I23 | Certain current complications following ST elevation (STEMI) and non-ST elevation (NSTEMI) myocardial infarction (within a 28-day period) |
| Angina | I20 | Angina pectoris |
| CS | I25 | Chronic ischemic heart disease    **Exclude I25.3 & I25.4 |
| ACS | I70 - I79 | Diseases of Arteries, Arterioles, and Capillaries |
| PAD | I70 | Atherosclerosis |
| PAD |  |  |
| PAD | I73.9 | Peripheral vascular disease, unspecified |
| PAD | I74 | Arterial embolism and thrombosis |
| PAD | I75 | Atheroembolism |
|  |  | Other CVD related diseases |
| MI |  |  |
| TIA | G45 | Transient cerebral ischemic attacks and related syndromes |
| PAD | I99 | Other and unspecified disorders of the circulatory system |
| PAD  PAD | I99  Z86.7 | Other and unspecified disorders of the circulatory system  Personal history of diseases of the circulatory system |
|  |  |  |
| Revascularization | Z98.6 | Angioplasty status |
| Revascularization | Z98.6  Z95.1 | Angioplasty status  Presence of cardiac and vascular implants and grafts |
| Revascularization | Z95.5 | Presence of coronary angioplasty implant and graft |
| Revascularization | Z95.8, Z95.9 | Presence of other cardiac and vascular implants and grafts |

| Comorbidities | |
| --- | --- |
| Diagnosis | ICD-10 |
| Congestive Heart Failure (CHF) | I50, I50.1, I50.2, I50.3, I50.4, I50.8, I50.9, I11.0, I13.0, I13.2, I42.0 |
| Hypertension (HTN) | I10, I11, I12, I13, I15, I16 |
| Diabetes Mellitus (DM) | E10.0, E10.1, E10.9, E11.0, E11.1, E11.9 |
| History of Stroke /TIA/ thromboembolism: | |
| - Stroke | I63, I64 |
| - TIA | G45 |
| - Thromboembolism (Systemic Embolism) SE | I74 |
| Vascular disease (Prior MI, peripheral arterial disease, or aortic plaque): | |
| - MI | I21, I22, I23, I25.2 |
| - Peripheral Arterial Disease (PAD) | I70.2 – I70.9; I71; I73.9 |
| - Aortic Plaque | I70.0 |
| Chronic kidney disease (CKD) Level: | |
| - Stage 1 with normal or high GFR (GFR > 90 mL/min) | N18.1 |
| - Stage 2 Mild CKD (GFR = 60-89 mL/min) | N18.2 |
| - Stage 3 Moderate CKD (GFR = 30-59 mL/min) | N18.3 |
| - Stage 4 Severe CKD (GFR = 15-29 mL/min) | N18.4 |
| - Stage 5 End Stage CKD (GFR <15 mL/min) | N18.5, N18.6 |
| - Unknown Stage CKD | N18.9 |
| Abnormal Renal Disease: | I12, I13, N00, N01, N02, N03, N04, N05, N07, N11, N14, N17, N19, Q61 |
| Abnormal Liver Disease | B15, B16, B17, B18, B19, K70, K71.1, K71.3, K71.4, K71.5, K71.6, K71.7, K72, K73, K74, K76.0, K76.2, K76.4, K76.5, K76.6, K76.7, K76.8, K76.9 |
| Hyperlipidemia | E78 |
| Cancer: | |
| - Solid Tumors | C00 – C80 |
| - Leukemia | C91-C96 |
| - Lymphoma | C81-C88 |
| - Metastatic cancer | C77, C78, C79 |
| Alcohol Use | F10, G62.1, I42.6, K29.2, T51, Z71.4 |
